# Drug choice, spatial distribution, HIV risk, and HIV prevalence among injection drug users in St. Petersburg, Russia

**DOI:** 10.1186/1477-7517-6-22

**Published:** 2009-07-31

**Authors:** Gina Rae Kruse, Russell Barbour, Robert Heimer, Alla V Shaboltas, Olga V Toussova, Irving F Hoffman, Andrei P Kozlov

**Affiliations:** 1 Baylor College of Medicine, Houston, TX, USA; 2 Center for Interdisciplinary Research on AIDS, Yale University School of Public Health, New Haven, CT, USA; 3 Department of Epidemiology & Public Health and the Center for Interdisciplinary Research on AIDS, Yale School of Public Health, PO Box 208034, 60 College Street New Haven, CT 06520-8034 USA; 4 The Biomedical Center, St. Petersburg, Russia and the Faculty of Psychology, St. Petersburg State University; 5 The Biomedical Center, St. Petersburg, Russia; 6 Department of Medicine, Division of Infectious Diseases, University of North Carolina, Chapel Hill, NC, USA; 7 The Biomedical Center, St. Petersburg, Russia; 8 Department of Medicine, Massachusetts General Hospital, Boston, MA, USA

## Abstract

**Background:**

The HIV epidemic in Russia has been driven by the unsafe injection of drugs, predominantly heroin and the ephedrine derived psychostimulants. Understanding differences in HIV risk behaviors among injectors associated with different substances has important implications for prevention programs.

**Methods:**

We examined behaviors associated with HIV risk among 900 IDUs who inject heroin, psychostimulants, or multiple substances in 2002. Study participants completed screening questionnaires that provided data on sociodemographics, drug use, place of residence and injection- and sex-related HIV risk behaviors. HIV testing was performed and prevalence was modeled using general estimating equation (GEE) analysis. Individuals were clustered by neighborhood and disaggregated into three drug use categories: Heroin Only Users, Stimulant Only Users, and Mixed Drug Users.

**Results:**

Among Heroin Only Users, younger age, front/backloading of syringes, sharing cotton and cookers were all significant predictors of HIV infection. In contrast, sharing needles and rinse water were significant among the Stimulant Only Users. The Mixed Drug Use group was similar to the Heroin Only Users with age, front/back loading, and sharing cotton significantly associated with HIV infection. These differences became apparent only when neighborhood of residence was included in models run using GEE.

**Conclusion:**

The type of drug injected was associated with distinct behavioral risks. Risks specific to Stimulant Only Users appeared related to direct syringe sharing. The risks specific to the other two groups are common to the process of sharing drugs in preparation to injecting. Across the board, IDUs could profit from prevention education that emphasizes both access to clean syringes and preparing and apportioning drug with these clean syringes. However, attention to neighborhood differences might improve the intervention impact for injectors who favor different drugs.

## Introduction

Injection drug use is at the heart of Russia's HIV epidemic and the majority of new infections are associated with injection drug use [1,2]. Of the approximately 40,000 new HIV cases registered in Russia in 2003, 76% were among injection drug users [3]. Heroin and psychostimulants are the dominant injection drugs of abuse in Russia [1,4]. St. Petersburg has been one of the most affected cities with nearly 40,000 reported HIV infections [5,6].

Among St. Petersburg IDUs, the type of drug injected is associated with incidence and prevalence of HIV infection. As previously reported, in a sample of drug users recruited in 2002 and followed for a year, psychostimulant use was associated with HIV incidence [7] while HIV prevalence was spatially clustered with frequent heroin use [8]. The behavioral effects of different drug types could have accounted for these differences. Outside of Russia, psychostimulant use has been associated with both HIV risk due to sharing injection equipment and an increase in HIV cases [9,10]. Historically, psychostimulants used in Russia are 'vint' and 'jeff'. These are home-made injectable derivatives of ephedrine, pseudoephedrine, or phenylpropranolamine (PPA). They cause amphetamine like effects with release of dopamine and serotonin and inhibition of dopamine and serotonin transporters after multiple administrations [11,12]. The stimulant effects have behavioral consequences including impulsivity, increased sexual activity, and injection risk taking including bingeing.

The high prevalence of stimulant use has been a significant concern in the fight against HIV. There are an estimated 35 million amphetamine type stimulant users worldwide [13,14], the second most widely used illicit drug after cannabis (161 million users). Opiates (16 million users including 11 million heroin users) remain the leading problem drug as measured by treatment demand. Opiate users constitute the majority of IDUs and understanding which behaviors put them at risk for HIV is a crucial component in fighting new infections. The situation in Russia is similar to patterns seen worldwide [1,15].

The goal of the present study was to compare high risk injecting practices between injection heroin users, stimulant users, and mixed drug users. A cohort of IDUs was recruited into the NIH, HIV Prevention Trials Network (HPTN) 033 HIV Prevention Preparedness Study, a multicentre study whose primary objective was to estimate rates of HIV seroincidence among persons who could participate in future HIV prevention studies. The incidence rate was 4.5/100 person years [7] with stimulant use being the strongest correlate to HIV acquisition. Secondarily, the study sought to describe the characteristics and risk behaviors of the screened cohort. HIV prevalence among this study cohort was 30% placing it among the worst epidemics among IDUs in Europe [16].

Gathering information on HIV infection and risk behaviors is necessary to focus interventions appropriately. The ultimate goal in studying this vulnerable population is to gain practical information that can be used to reduce HIV transmission by means of public health interventions; toward this end, we identified risks typical to different substance use patterns, information which can inform prevention efforts.

## Methods

### Participant Recruitment

Active IDUs were recruited over a 10 month period by peer recruitment, street outreach and from rehabilitation and detoxification facilities. Details of the recruitment patterns [16] and spatial distribution of participants' place of residence that have been reported previously [8] demonstrated that the sample was broadly distributed throughout the city of St. Petersburg, to some extent overcoming limitations imposed in a non-random sampling if only a single recruitment method had been employed. Individuals were eligible if they injected drugs at least three times per week in the previous month or if on at least three occasions in the previous three months they used injection equipment after another person. Active injection was assessed through detection of recent injection stigmata. Individuals with apparent psychiatric disorders were excluded. Initially, individuals 18 years or older were recruited but this was expanded to include those 16 years or older near the end of the recruitment period. Institutional Review Boards (IRBs) at The Biomedical Center and the University of North Carolina approved the study before it started as well as the change in protocol that lowered the age of consent. Additionally, a community advisory board was developed in St. Petersburg for the purpose of ensuring that participants' rights were protected. Screening was conducted as part of the HIV Prevention Trials Network (HPTN) 033 study designed to enroll a cohort of seronegative IDUs for a year's follow-up study preparatory to initiating prevention in St. Petersburg Russia.

The sample accrued for this report consisted of all screened individuals, regardless of HIV serostatus. The time of seroconversion for all HIV positive individuals could not be determined, so the sample must be considered a mix of seronegative and seroprevalent individuals.

### Data Collection

After giving informed consent, individuals completed baseline questionnaires that collected data on sociodemographics, drug use, place of residence and injection- and sex-related HIV risk behaviors and were tested for HIV [16]. Questions on drugs injected and injection practices covered the three months prior to the interview whereas questions of sexual activity covered the six months prior to the interview. The data collection instrument was common to all four international sites participating in HPTN 033.

### Statistical Methods

In a previous analysis, taking into account the spatial distribution of study participants across the city, we were able to locate exact addresses for 788 of the 900 participants screened and we explored the co-clustering of HIV prevalence and variables from the questionnaire using the Moran's I statistic and the Nearest Neighbor algorithm [8]. In the current analysis, we sought to overcome the loss of statistical power from the elimination of 112 observations in the spatial analysis while adjusting for the statistically significant clustering. Therefore, we applied generalized estimating equations (GEE) using the participant's neighborhood of residence as a clustering factor to capture the effects of the previously observed spatial correlation. For this analysis, individual point data were aggregated by residential districts using ESRI ArcMap GIS software with the "HawthTools" extension. One subject could be included in this analysis for a total of sample of 899. Participants identified sixteen discrete neighborhoods in which they resided; the thirteen within the city boundaries are included in the maps.

The sixteen neighborhood grouping proved to be consistent with the results of the purely spatial analysis in that risk clusters generated ellipsoids that generally followed residential neighborhoods, making this a rational aggregating factor for GEE analysis. Given the dichotomous nature of the dependent variable, HIV prevalence, we applied logistic regression within GEE as suggested by Shaw et al. [17].

Three software programs were necessary to achieve a robust statistical analysis, due to the spatial structure of the data and the differing capabilities of each program. It should be noted that the statistical models presented in this paper were consistent across all three software programs we applied, with the variables listed as significant or not significant remaining so, despite small variations in standard errors. However, to maximize the robustness of the analysis, we felt compelled to select different software for different applications as follows. An initial run of preliminary statistical models on ***Splus ***7.0 and the xtgee command in STATA 9.2 suggested that accounting for clustering by neighborhood under a GEE would unmask additional relationships between HIV and demographic and behavioral variables. Our variable reduction strategy tested for univariate associations individually by logistic regression within GEE as suggested by Shaw et al [17]. In contrast to Shaw et al, we applied a stricter criterion of *P *< 0.10 for the resultant Wald statistic, versus *P *< 0.20 for candidate variables. Candidate variables were then entered into a multivariate model again using the logistic regression within the GEE framework of the ***STATA xtgee ***command. Variables not significant at the *P *< 0.05 were eliminated with the exception of an education level variable and a housing variable, since they did not seem relevant to possible harm reduction strategies.

Shaw et al. also note that parameter estimation in GEE is through quasi-likelihood [17]; therefore, standard model selection criteria such as stepwise techniques and the Akaiki Information Criteria (AIC), which are based on likelihood methods, were not appropriate. We therefore applied the Quasi-likelihood Information Criteria (QIC) as proposed by Pan calculated in a module developed for STATA software by Cui for variable reduction and model selection [18,19]. Since data were collected from only sixteen neighborhoods in St. Petersburg – less than the thirty clusters that are usually required for GEE – we accounted for the low number by using a "jackknife" standard error as recommended by Hardin and Hibble (2003). "Jackknife" standard errors are not available in ***Splus***, so the analysis was re-run using the GEE algorithm in the ***R geepack ***software package add-on developed by Yan and colleagues [20,21]. Significance levels in this algorithm are based on the Wald statistic.

Finally, we disaggregated the data by current drugs injected creating three distinct categories of drug user based on the drug(s) injected in past 30 days: heroin only users, stimulant only users, and mixed drug users.

## Results

We included data from a total of 899 recruited individuals. As previously reported, the sample was 71% male with a median age of 24, four in five had completed secondary education and half had some post-secondary education, 43% of the sample was unemployed at the time of interview, only 17% was living in a residence that they owned or rented; and 30% was confirmed HIV seropositive [16]. As noted in Table 1, 430 (48%) reported heroin use only, 30 (3%) stimulant use only, and 439 (49%) mixed drug use. All those in the mixed drug use category injected had injected both heroin and psychostimulants in the three months prior to interview; 76 people (17% of those in the mixed drug use category) reported injecting other drugs. The three groups did not differ in their demographic characteristics.

**Table 1:** HPTN Drug Use Distribution

**Drug Use**	**Count**	**% of Total**
Heroin Only	430	47.8
Stimulants Only	30	3.3
Mixed	439	48.8
Total	899	

**Figure 1:**
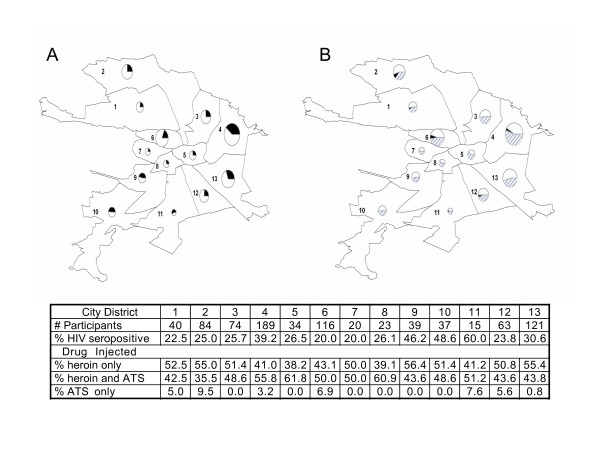
**Spatial Distribution of HIV Cases and Injection Drugs within the City of St. Petersburg**. Data from 899 participating injection drug users screened at baseline were sorted by district of residence. For each district, the number of participants, the HIV prevalence, and the percentage of injectors who injected only heroin, only amphetamine-type psychostimulants (ATS), or both within the 30 days prior to entering the study are included on the embedded table. The maps display HIV prevalence (A) and type of drug injected (B) with the size of the pie charts proportional to the number of participants who resided in each of the 13 neighborhoods within the city limits of St. Petersburg. For HIV prevalence, the dark part of the pies represents the seropositive cases. For drug injected, the open part of the pies represents heroin only injectors, the dark part represents ATS only injectors, and the striped part represents injectors of both kinds of drug.

Spatial analysis at the district level found levels of HIV prevalence that ranged from 20% to 60% (mean = 31.9% ± 12.6%, median 26.1%) with minor positive skewness (Figure 1A). Spatial analysis also revealed that stimulant only users resided in only seven of the city's thirteen districts, but they did not appear to be concentrated in spatial contiguous districts (Figure 1B).

For the sample as a whole, the choice of drug injected did not predict differences in injection frequency, behaviors, or practices. Conversely, neighborhood alone in the absence of the inclusion of the type of drug used did not reveal significant associations between HIV prevalence and risk behaviors. However, when we adjusted for location of residence using GEE and looked specifically at the behavioral attributes associated with HIV infection we could detect significant differences among users of different drugs (Table 2). The GEE models of HIV prevalence among heroin only users revealed that younger age, front- or back-loading, sharing cotton, and sharing cookers were significant. The same variables, with the exception of sharing cookers, were significant among the mixed drug users. The stimulant only users were different from the other injectors in two injection risk categories. Those who were HIV positive within the group were more likely to engage in receptive needle sharing but were less likely to share rinse water.

**Table 2:** GEE Wald Statistic *P *values of Unsafe Injection Behaviors Related to HIV Prevalence by Drug Use

Variable	Heroin Only	Mixed Drug	Stimulant Only
Younger Age	**0.012**	**<0.001**	0.784
Share with front- or back-loading	**0.007**	**<0.001**	0.754
Receptive syringe sharing	0.252	0.082	**0.017**
Sharing cotton	**0.045**	***<0.001***	0.092
Sharing cooker	***0.014***	0.098	0.456
Sharing rinse water	0.188	0.118	***=0.001***

*P values in **bold **are significant; *italics *are negative relationships

## Discussion

These data revealed that drug users in St. Petersburg who inject only stimulants and live in certain neighborhoods appear to constitute a unique population in terms of HIV exposure risk, even though their proportion in the sample is small. Almost all (97%) drug users in the cohort injected heroin (either alone or in combination with or in addition to stimulants) while only a small number injected stimulants only. Stimulant users did not differ demographically from the heroin users as a whole, but the risk behaviors associated with HIV infection did when considered in the context of neighborhood of residence. Whereas front or back-loading and sharing non-syringe equipment were significantly associated with HIV infection among heroin users (either those who injected only heroin or both heroin and stimulants), receptive syringe sharing was significant among the stimulant only users.

Studies conducted both in the former Soviet Union and elsewhere in the world have reported differences in risk behavior between stimulant injectors and opioid injections [22-24]. A study of risk behaviors by type of drug used in Ukraine found front and back-loading was more common among opiate injectors while reusing a syringe was more common among stimulant users [25]. While heroin has been associated with passivity, regular injection and decreased sexual activity, stimulant use has been associated with aggression, frequent, binge injection, needle sharing, increased sexual activity and young age [26]. It has been suggested that contrasting physiologic responses to opiates versus stimulant drugs result in different risk profiles for HIV [11,27-29]. For St. Petersburg, this is supported by the observation that frequent stimulant use is the primary factor associated with seroconversion [7]. The present analysis reveals that even though stimulant users share demographic and behavioral characteristics with heroin users, their behavior distinguishes them in terms of HIV risk as ascertained by prevalence rates.

Our data are subject to limitations. First, the number of injectors who used only stimulants was quite small. Given that injection of stimulants only is unusual among drug users in Russia as a rule, increasing the sample size is unlikely to yield many additional such individuals [1,15,30]. Second, associations between risk and prevalent HIV infection were revealed only when correlation by residence was included in the analysis, which suggests that geographic differences in risk may be as important as differences in the type of drug injected. Further research will be needed to determine if the choice of drug remains a significant factor in predicting transmission of HIV among drug injectors in St. Petersburg. Third, while our sample appears to be representative of drug users in St. Petersburg and is distributed randomly across the city [8], the results may not be generalizable to populations outside of St. Petersburg. However, if characteristic effects and preparation processes for the different drugs explain some of our observed behavioral differences then the differences could occur among drug users in other settings. Fourth, our data analysis permitted us to identify associations that link prevalent, but not incident cases of HIV to drug type, injection risks, and geography. It must be noted however, that when we followed 520 seronegative individuals in our sample for an additional year, we found that psychostimulant use was strongly associated with incident infections, with hazard ratios of 8.1 for individuals who made three or more psychostimulant injections weekly versus those who made none [7] and 5.5 for psychostimulant only injectors versus heroin only injectors. However, no injection practices were associated with incidence, a consequence of the low power provided by the smaller number of psychostimulant injectors in the seronegative cohort. These findings lead to the hypothesis that there is an association between receptive syringe sharing, which was more common among the psychostimulant only injectors, and HIV transmission, but more research would be needed to test this hypothesis. Finally, needle and syringe sharing is a widely recognized risk factor for parenteral infections and may be more socially unacceptable than sharing other drug preparation equipment. This could result in a socially desirable response bias leading to under-reporting of needle and syringe sharing compared with other equipment sharing behaviors. However, this under-reporting would not account for the association of stimulant injection, receptive syringe sharing, and HIV prevalence in one small subset of the population while failing to find such an association in a larger subset given the statistical power of the larger subset.

The role of geography was evident in our findings, but its exact impact was hard to determine. Since psychostimulant only injection was associated with certain city districts, with receptive syringe sharing, and with subsequent seroincidence [7], it seems likely that the interaction of a risky injection practice with districts in which HIV prevalent cases were already clustered [8] is sufficient to explain the increased likelihood of HIV transmission among psychostimulant injectors. The one district with both incident infections and psychostimulant injection was (and remains) a fairly typical residential district of apartment blocks connected to the rest of the city by bus, metro, and rail. Since little neighborhood ethnography has been conducted to study local variations in the drug scene across districts in St. Petersburg, it is hard to speculate on neighborhood contextual factors that might have further enhanced risk for injectors who resided there.

In conclusion, differences in drug preparation and distribution practices for opioid versus stimulant injection may account for some differences in risk and exposure to HIV and other bloodborne viruses [4,31]. Some of these differences may be reflected in the spatial component of our findings – that neighborhood of residence is an important covariate when studying the relationship between HIV prevalence and risk behaviors. In designing targeted interventions, it becomes important to address both the drug type and the neighborhood differences since they result in distinct routes of infection. More generally, intervention programs to reduce HIV among this population should identify and focus on risk behaviors specific to the type of drug used and the social context in which is it used [32]. Across the board, IDUs could profit from prevention education that emphasizes both access to clean syringes and preparing and apportioning drug with these clean syringes, but slight differences in emphasis and attention to neighborhood differences might improve the intervention impact for injectors who favor different drugs.

## Competing interests

The authors declare that they have no competing interests.

## Authors' contributions

Drs. Barbour and Kruse contributed equally to the drafting and revising of the manuscript. Dr. Kruse began the data analysis and Dr. Barbour provided the analytical acumen to recommend the application of GEE to the data. Dr. Heimer and Dr. Kozlov supervised the manuscript preparation. Drs. Shaboltas and Toussova led the participant recruitment, collected the data, and maintained the pariticpant database. Drs. Shaboltas supervised the day-to-day work of Dr. Kruse while she was on-site in St. Petersburg and Drs. Heimer and Kozlov were overall mentors for Dr. Kruse during her year in Russia. Drs. Kozlov and Hoffman were co-principal investigators on the HPTN-supported study from which the current manuscript draws the baseline data and Dr. Heimer contributed to the design of participant recruitment. All authors read and contributed editorial suggestions to the manuscript during the iterative process of moving from first draft to submitted form.
